# Comparing the language style of heads of state in the US, UK, Germany and Switzerland during COVID-19

**DOI:** 10.1038/s41598-024-51362-7

**Published:** 2024-01-19

**Authors:** Olenka Dworakowski, Tabea Meier, Matthias R. Mehl, James W. Pennebaker, Ryan L. Boyd, Andrea B. Horn

**Affiliations:** 1 URPP “Dynamics of Healthy Aging”, University of Zurich, Zurich, Switzerland; 2 Department of Psychology - Gerontopsychology, University of Zurich, Zurich, Switzerland; 3 Department of Psychology, The University of Texas at Austin, Austin, USA; 4 Department of Computer Science, Stony Brook University, New York, USA; 5 Department of Psychology, University of Arizona, Tucson, USA; 6 Competence Center Gerontology, University of Zurich, Zurich, Switzerland

**Keywords:** Psychology, Human behaviour

## Abstract

The COVID-19 pandemic posed a global threat to nearly every society around the world. Individuals turned to their political leaders to safely guide them through this crisis. The most direct way political leaders communicated with their citizens was through official speeches and press conferences. In this report, we compare psychological language markers of four different heads of state during the early stage of the pandemic. Specifically, we collected all pandemic-related speeches and press conferences delivered by political leaders in the USA (Trump), UK (Johnson), Germany (Merkel), and Switzerland (Swiss Federal Council) between February 27th and August 31st, 2020. We used natural language analysis to examine language markers of expressed positive and negative emotions, references to the community (we-talk), analytical thinking, and authenticity and compare these language markers across the four nations. Level differences in the language markers between the leaders can be detected: Trump’s language was characterized by a high expression of positive emotion, Merkel’s by a strong communal focus, and Johnson’s and the Swiss Federal Council by a high level of analytical thinking. Overall, these findings mirror different strategies used by political leaders to deal with the COVID-19 pandemic.

## Introduction

The COVID-19 pandemic was a historically and uniquely challenging time in several ways. Not only did the pandemic pose a threat to our physical health, but also to our social life, sense of security, and mental health^1^. Political leaders were tasked with safely guiding people through a worldwide crisis. The decisions of political leaders affected the public in more direct and measurable ways than usual: health measures and restrictions (e.g., lockdowns, social distancing) disrupted people’s daily lives in ways that many had never before experienced. Moreover, such measures were implemented in a “top-down” fashion, with little consensus-building or advance notice, which is particularly uncommon in democratic societies. Especially during the beginning of the pandemic, when uncertainty was at its highest, political leaders had to communicate drastic public health measures while also trying to convey some sense of safety and optimism.

Public speeches and press conferences are the most direct way for political leaders to connect with the public at large. The public gravitated towards these events and thedirect contact and guidance from their leaders: For example, the first televised address made by Angela Merkel at the beginning of the pandemic on March 18, 2020, was watched by over 25 million Germans—almost one-third of the German population^2^. Merkel’s speech was delivered shortly before Germany introduced its first lockdown measures. Nations around the world had similar moments, where heads of state introduced measures that impacted people’s daily lives in unprecedented ways. The global pandemic constitutes a historically outstanding moment during which people around the world paid particular attention to their leaders.

The aim of this research is to describe and compare the language style of these political leaders, in order to investigate this behavioral measure of leadership and communication in times of crisis. This research is embedded in current views on the social psychology of language use^3–5^. The social psychology of leader statements during the initial phase of the pandemic opens a unique window for researching the diverse response styles of leaders during a similar situation of crisis. How did different political leaders communicate and what kind of leadership did they provide?

There is a long-standing tradition of understanding society-level processes through the language of political leadership^6–8^. Early language analysis methods were demonstrated to be a valuable tool for unveiling and investigating political leaders’ motivations and success^9,10^. With recent technological progress, researchers can apply automated text analysis methodsto investigate long-term developments of political leader language at scale^3^. Particularly in times of crisis, leadership language, by its very nature, provides insight into relevant motivational, emotional, and ideological facets^11,12^. The COVID-19 pandemic represents a moment of crisis wherein the words used by heads of state and public institutions affected the daily life of populations^13,14^.

While heads of state across multiple nations often issued similar measures, and thus communicated comparable content to their citizens, they did so in remarkably different ways. Previous studies have shown that stylistic aspects of political leaders’ language influence citizens’ collective responses^15^. The current research investigates the language styles of the heads of state of four different nations during the first months of the COVID-19 pandemic. Specifically, we focus on the USA, UK, Germany, and Switzerland to describe and compare the language styles of Donald Trump, Boris Johnson, Angela Merkel, and the Swiss Federal Council (FC) between February and August 2020. A comparison of these 4 nations is empirically worthwhile as they share many similarities at the level of the nation whereas, at the level of their political leadership, their heads of state during the pandemic differed substantially in their leadership styles and political orientations. At the nation level, all of them can be considered WEIRD (Western, Educated, Industrialized, Rich, Democratic) cultures with federalist political systems and comparable cultural values (e.g., relatively low values in power distance, high levels in individualism, and masculinity; https://www.hofstede-insights.com/). The heads of state (during the pandemic), however, differed in both their political views^16^ and communication styles^17–20^. Trump, for example, is known for his authentic, grandiose language^21,22^, while Angela Merkel is known for her focus on the community^19^.

The COVID-19 pandemic further provides a situational similarity in terms of what the nations were going through. All 4 nations reported their first case between January and February 2020. All nations introduced similar health measures, finally initiating a lockdown in March 2020. Additionally, each nation began relaxing measures in the Spring of 2020, and the case numbers also showed similar increases during the first few months and then decreased after the lockdowns. The cases decreased less in the USA and increased again later around June 2020, while the cases in the other nations remained relatively low. All in all, the situations were relatively similar in the nations during this period, with the pandemic spreading in these nations for the first time and all initiating similar measures.

When giving a speech, political leaders convey more than just the content they are communicating—the *style* of a speaker’s language, as reflected by their use of function words (e.g., articles, pronouns, conjunctions, etc.) have been shown to be powerful markers of both discourse and psychological processes. State-of-the-art automated language analysis tools allow us to analyze political speeches and characterize them along psychologically meaningful dimension^4,23^. Through the words they use, political leaders unveil a wealth of information about themselves, ranging from insights into their personality and thinking styles to how authentic they are^4^. To assess dimensions of interest in a psychometrically sound way, the Linguistic Inquiry and Word Count (LIWC) approach has a demonstrated reliability and validity in this research domain. For example, research shows that individuals who use higher rates of articles and prepositions tend to engage in more complex and abstract thinking^23^. As another example, using more first person plural pronoun “we” reflects an individual’s communal orientation and bonding^24^.

Here, we briefly introduce the language markers of LIWC included in this study, providing an overview of why these measures are important in the context of early pandemic communication by political leaders. To date, first-person plural pronouns (we-talk), positive and negative emotional tone, analytical speech, and authenticity have been identified as meaningful dimensions of political leader language; they have been identified to reflect communal orientation, the emotional tone and cognitive complexity of the message conveyed, and how authentically the person is speaking^3,4,22,25^. In the following, we will shortly introduce these dimensions and corresponding relevant studies.

### We-talk

A higher use of first personal plural pronouns (e.g., we, us, our) by political leaders communicates a focus on communal processes and solidarity^11,26^. Both political leaders^11^ and individuals^27^ have been shown to use more we-words during times of crisis, thus indicating a shift in mindset from the individual to the collective. We-talk by political leaders during the pandemic has also been associated with less anxiety and sadness as well as more positive responses from the general public^28^.

### Emotional language

Emotion words convey a speaker's focus on either positive or negative emotions, as well as more general emotional processes^29^, conveying a sense of optimism or pessimism regarding a situation. Emotional language is often part of a politician’s strategy in campaigning, for example, by leveraging positive emotion language to appeal to the public (e.g., use enthusiasm to attract or percuade voters;^30^, and populist leaders in particular often try to evoke especially strong emotions in the population^31^. During the pandemic, political leaders might have tried to convey a positive attitude in the hopes of up-regulating the public mood through processes of emotional contagion^32^. In previous studies, negative emotions expressed by political leaders during the pandemic were linked to citizens’ more communal focus on a collective level^28^.

### Analytical thinking

In addition to investigating we-talk and emotional tone, we can gain insight into the ways people think by analyzing other stylistic aspects of the language, such as articles and prepositions. People who portray a more narrative and intuitive way of thinking tend to use more pronouns, adverbs, and auxiliary verbs, while more analytical, logical thinkers tend to use more articles and prepositions in their speech^23^. Analytical thinking, measured in language, has been shown to correspond to greater academic performance and cognitive elaboration^23,33,34^. In a political context, analytical speech has been found associated with electoral success in US-American presidential elections in 2003–2004^35^ and 2020 US Congress elections^36^, suggesting that analytical speech conveys more competence. Since then, there have also been studies showing a decrease in analytical language in US political leaders over time^3^. In previous studies, we have found that analytical speech of political leaders during the pandemic to be associated with more positive emotions as well as communal focus in the collective public^28^.

### Authenticity

Lastly, past research demonstrates that a speaker’s authenticity is reflected in specific patterns of language use. Section “Authenticity” refers to the degree to which a person is communicating in a relatively spontaneous, unfiltered manner^5^, and is reflected in greater use of self-referential, more complex language (i.e., language focusing more on evaluation and judgments than simple, concrete language) and less distancing^25^. Importantly, authenticity is distinct from honesty. People can communicate authentically but not tell the truth^5^. Authentic language can be described as a free and natural expression of one’s ideas^37^. Less authentic language would make a political leader seem more deceptive, evasive, or distant^22^. Authenticity could be particularly important during a pandemic for people to feel more connected and closer to their political leader.

This paper aims to describe and directly compare these specific linguistic features of Trump, Johnson, Merkel, and the Swiss FC during the beginning of the COVID-19 pandemic. First, we quantify the words most used by these representatives of executive power. Then, we investigate whether and how Trump, Johnson, Merkel, and the Swiss FC differ in their communication style in terms of analytical speech, authenticity, emotional tone, and communal orientation, given their relevance to understanding political discourse^4^. We expect different approaches, strategies, and attitudes toward the pandemic to be reflected in linguistic features, providing exemplars at relevant time points.

## Methods

### Data

**Figure 1:**
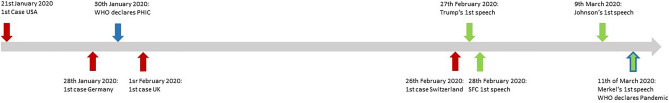
Timeline. (*Note*. The red arrows indicate the first documented infections of COVID-19 in each nation. The green arrows indicate the first speeches of each head of state and the blue arrows indicate a world-wide event relating to the WHO).

We collected transcripts of all speeches and press conferences concerning the COVID-19 pandemic of Donald Trump, Boris Johnson, Angela Merkel, and the Swiss FC from their first speech about the COVID-19 pandemic until August 30th, 2020. The dates of the first speeches were as follows: February 27, 2020 (Donald Trump), March 9, 2020 (Boris Johnson), March 11, 2020 (Angela Merkel) and February 28, 2020 (Swiss FC). We included all public speeches and press conferences concerning the COVID-19 pandemic these heads of states held during our time frame. Figure 1 provides an overview of the timeline of speeches and critical events in the 4 nations. During his press conferences, Boris Johnson did not answer questions for the audience; consequently, for all the other political leaders we retained only their language delivered before interactions with audience members to prevent the potential confound of format differences. For Trump, Johnson, and Merkel, transcripts were all publicly available online. For the Swiss FC, only videos were publicly available on YouTube; these press conferences were manually transcribed. In Switzerland (which has 4 official languages), the press conferences were held mostly in German, but parts were also spoken in French or Italian. The French and Italian parts were translated by a simultaneous translator in real-time during the press conference. We also transcribed these translated parts and included them in our analysis, meaning we used the entire German texts. Previous studies have shown that translators successfully preserve the linguistic style of the original speech^38^.

As the Swiss head of state is not occupied by one person, as in e.g., a presidential system, but collectively, we decided to use the collective language signature of the Swiss FC. The Swiss FC constitutes the branch of the highest executive power in the Swiss political system. The 7 members of the Council are also ministers and share the power collectively (see https://www.admin.ch/gov/en/start.html for more information). All decisions are made jointly and the Federal Council always appears united. For these reasons, we considered the speech of all Federal Council members jointly.

All texts were analyzed with the English or German versions of LIWC2015, depending on language of the text^39,40^. LIWC operates by categorizing words within a text into different psychologically meaningful categories. The resulting values are the percentages of these words with respect to the whole text, and in some cases, composite scores of different combined categories (e.g., analytical thinking, authenticity, see below). LIWC has been extensively used in research and the equivalence of the German with the original English^40^ version has been empirically established^38,39^. We used LIWC2015 since it is the most recent version of LIWC that is available with psychometrically validated dictionaries for both languages. We used the default settings for LIWC2015 in the respective languages when running the analysis.

We consider the following categories of LIWC, which all have been previously, successfully employed and validated in the context of political leaders’ speeches, as described above: We used we-talk as an indicator of the communal focus of political leaders^11^. Further, we used the categories of positive and negative emotions as indicators of the emotional tone of the speeches^22^. We used the default LIWC dictionaries of emotional tone, as they have been developed and tested to derive indicators of positive and negative emotionality beyond content^41^. Moreover, we considered the LIWC category of analytical speech as an indicator of interpersonal distance, as well as competence and cognitive ability^33^. Lastly, we considered the category of authenticity as an indicator of connectedness with the public and less psychological distancing^5^.

### Analysis

The main aim of this paper is to describe the unique linguistic features of the different individual heads of states. First, to give insight into the essential contents of political speeches during the pandemic, we present word clouds of all the speeches of each leader (i.e., one cloud for each leader), offering a description and comparison of the distinct speeches. Word clouds are a visualization based on the frequency with which a word is used in a text, representing more frequent words with correspondingly larger font sizes, and excluding common stop words (i.e., words with no inherent meaning such as pronouns). in a larger font. Next, we conducted an analysis of variance (ANOVA) to compare each LIWC language marker and the overall word count between the political leaders. All analyses were conducted in R.

## Results

Donald Trump spoke on 62 days, Boris Johnson on 21, Angela Merkel on 25, and the Swiss FC on 23. The total word count was 161,559 for Trump, 25,183 for Johnson, 22,802 for Merkel, and 33,234 for the Swiss FC.

### Words most used by these representatives of executive power

**Figure 2:**
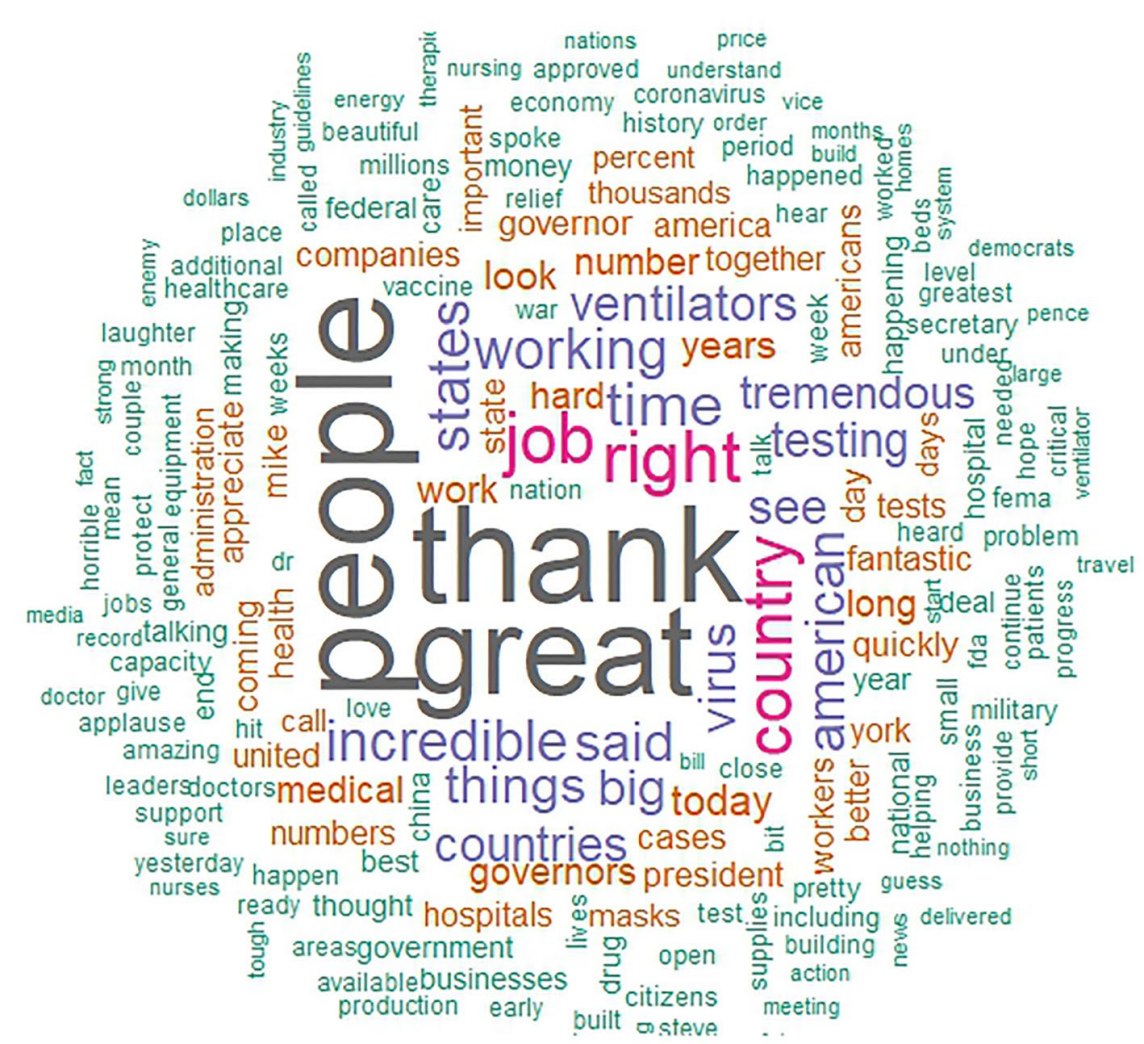
Word cloud of Donald Trump. (*Note.* Size of the words correspond to the frequency this word was said by Donald Trump).

**Figure 3:**
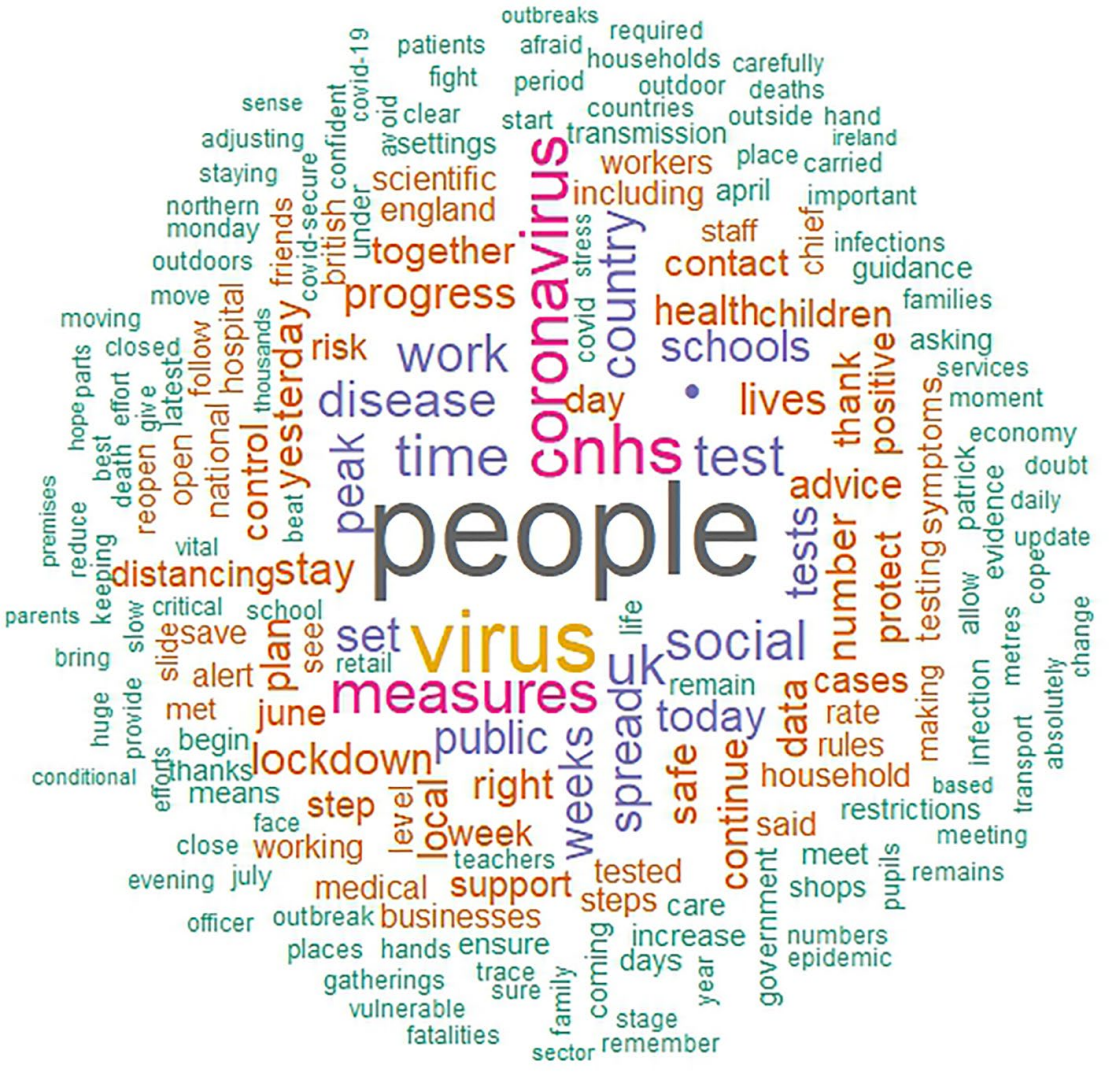
Word cloud of Boris Johnson. (*Note.* Size of the words correspond to the frequency this word was said by Boris Johnson).

**Figure 4:**
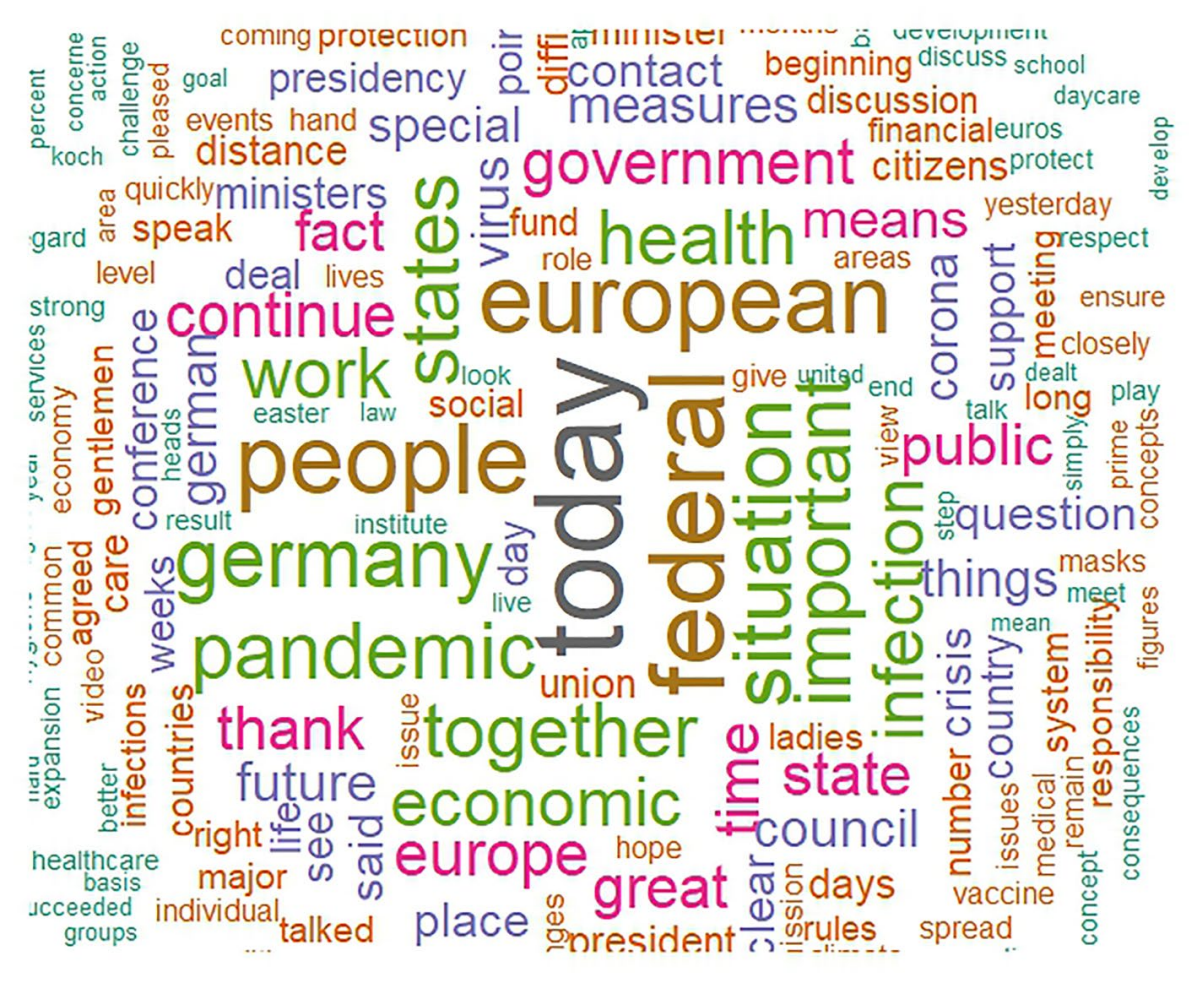
Word cloud of Angela Merkel. (*Note.* Size of the words correspond to the frequency this word was said by Angela Merkel).

**Figure 5:**
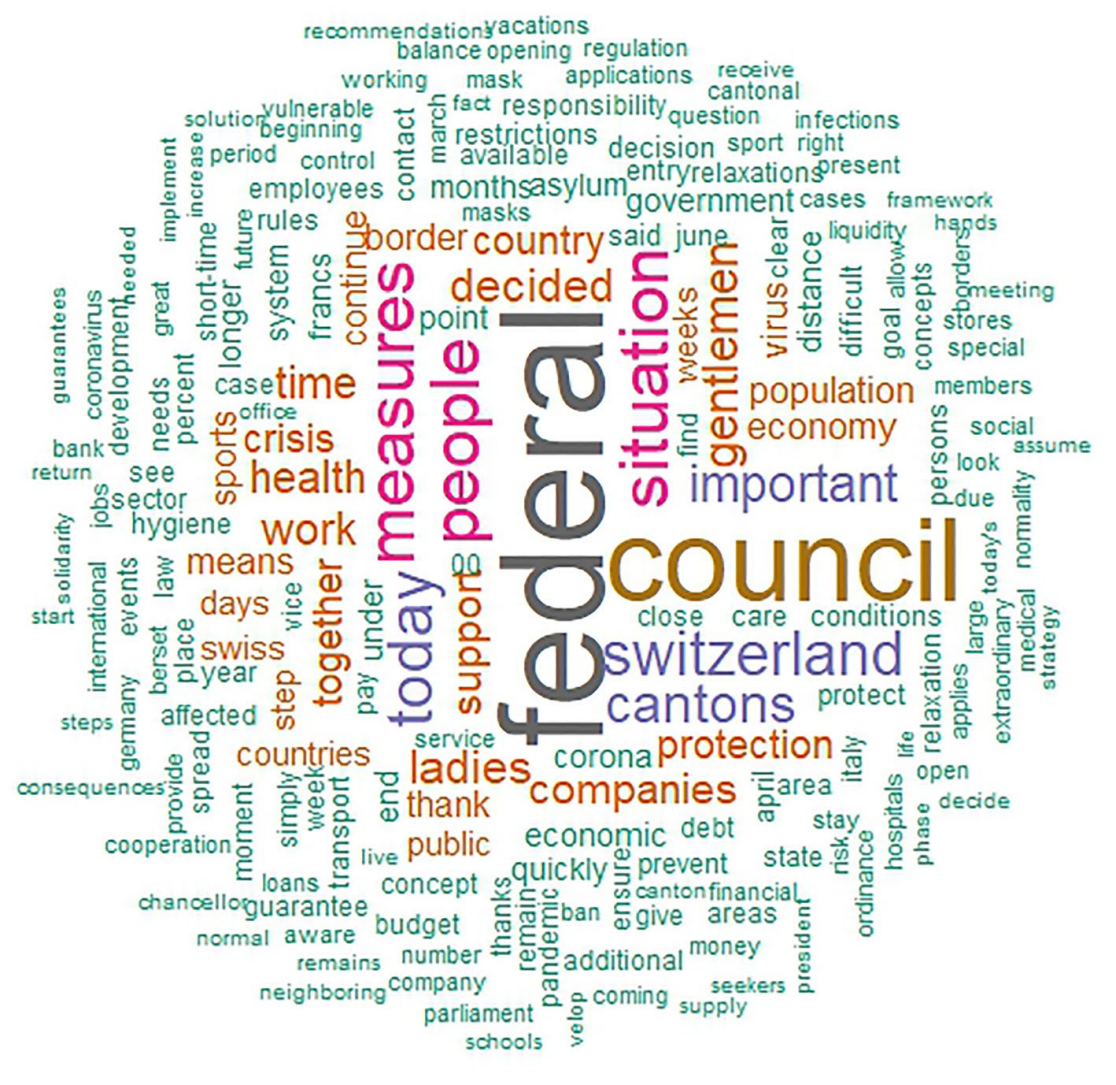
Word cloud of Swiss Federal Council. (*Note.* Size of the words correspond to the frequency this word was said by the Swiss Federal Council).

Figures 2, 3, 4, 5 show the word clouds of the most used words of each political leader. The word clouds suggest that both Trump and Johnson spoke a lot about ‘people’, consistent with the notion of them being considered populist politicians. However, Angela Merkel also mentioned ‘people’ a lot, despite not commonly considered to be a populist. Trump, moreover, also had ‘thank’ and ‘great’ as prominent words, hinting at the positive tone of his speeches. Johnson spoke at length about ‘virus’, ‘coronavirus’, and ‘measures’. This is likely a reflection of Johnson calling upon the public to help fight the virus. What is more, we also see ‘European’ as a popular word for Merkel, while the Swiss FC seem to mention themselves in the third person very often. This goes in line with them representing a collective council and standing behind their decisions as one.

### Language style differences between heads of state

#### Analytical speech, authenticity, emotional tone, and communal orientation

**Figure 6:**
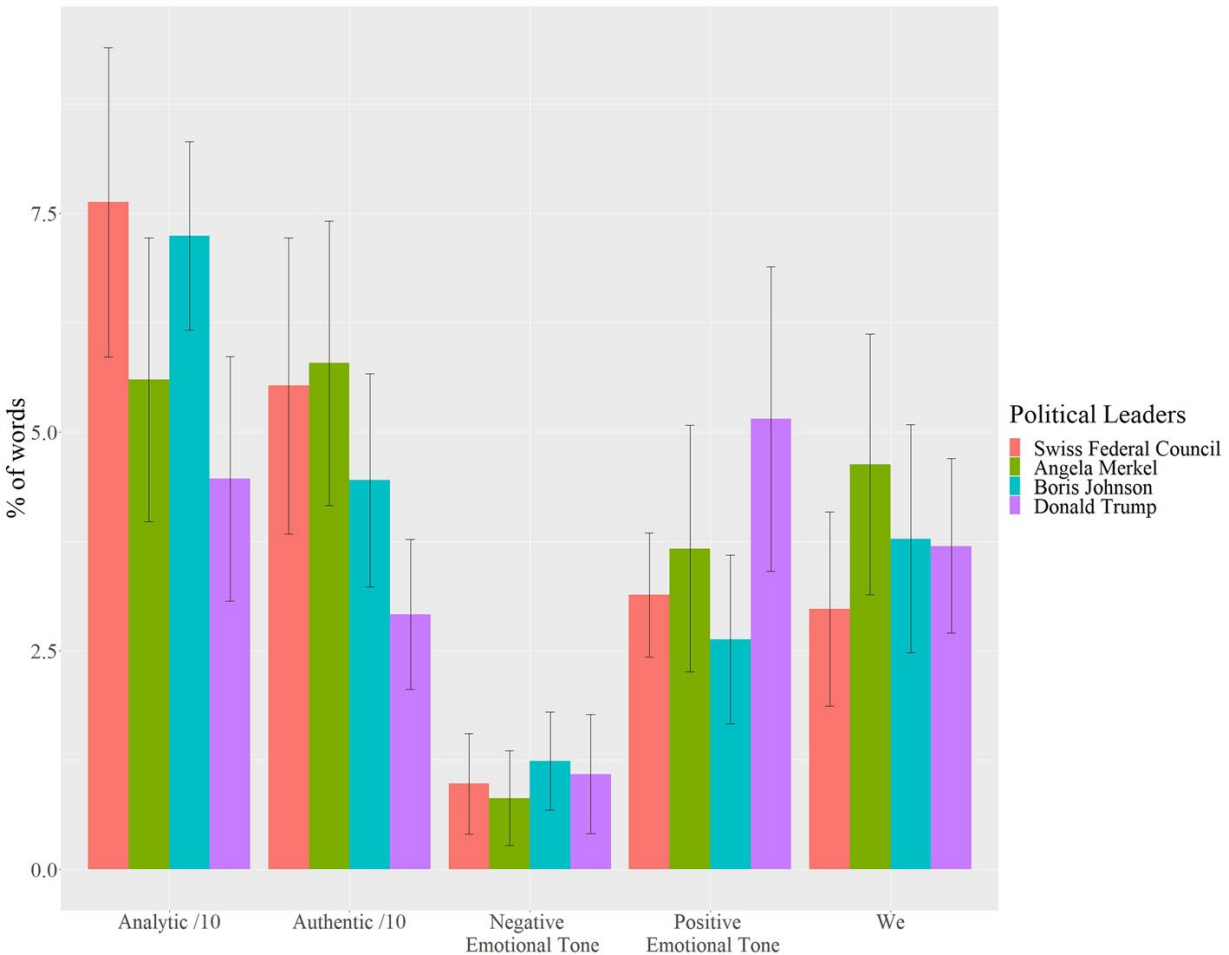
Means and standard deviations of language markers. (*Note*. The bars indicate the mean levels of each language marker and the lines indicate the standard deviation. Analytical speech and authenticity were divided by 10 for scaling.)

Figure 6 presents the means and standard deviations of the language features of each political leader. Table 1 summarizes the results of the analysis of variance of each language variable, it shows the differences of the means between each nation for each variable, with marked significant differences.

**Table 1:** Results of Tukey HSD Multiple Comparison of Means.

	Trump	Johnson	Merkel	Swiss FC	Differences
Analytic *M (SD)*	44.70 (14.10)	72.40 (11.00)	56.00 (16.60)	76.30 (17.70)	Swiss FC, Johnson > Merkel > Trump
Authentic *M (SD)*	29.20 (8.65)	44.20 (12.70)	57.90 (14.10)	55.30 (16.90)	Merkel, Swiss FC > Johnson > Trump
Negative Emotion* M (SD)*	1.10 (.54)	1.49 (.48)	0.90 (.47)	0.98 (.58)	Johnson > Trump, Swiss FC, Merkel
Positive Emotion *M (SD)*	4.72 (1.61)	2.71 (.54)	3.97 (1.28)	3.14 (.71)	Trump, Merkel > Johnson Trump > Swiss FC
We *M (SD)*	4.06 (1.02)	4.01 (.88)	4.88 (.90)	2.98 (1.11)	Merkel > Trump, Johnson > Swiss FC
Wordcount *M (SD)*	2606 (2062)	1199 (496)	912 (384)	1576 (857)	Trump > Swiss FC, Johnson, Merkel

M: mean, SD: standard deviation. Significant differences between political leaders are indicated by > , significance level is defined as *p* > .05.

### We-talk

Angela Merkel had the highest rates of ‘we-talk’ and the Swiss FC has the lowest. Trump expressed significantly less we-talk than Angela Merkel, but significantly more than the Swiss FC. Johnson also showed significantly less we-talk than Merkel and significantly more than the Swiss FC. Angela Merkel also showed significantly more we-talk than the Swiss FC.

Pennebaker and Lay^11^ describe different meanings of ‘we’. In specific sentences, ‘we’ might rather refer to ‘you’ (e.g. ‘we still have to take out the trash’), or mean a specific group. That is, we-words may not universally refer to ‘all of us’ as we might expect in a political leader’s speeches. Based on simply counting the frequency of the word “we,” it is not possible to differentiate between its various connotations. To explore the semantic context of the heads of states’ we-talk, we took a closer look at the exemplary speeches of Johnson and Merkel to investigate the meaning behind the communal focus.

On the 30th of April 2020, Boris Johnson said:

“We must be sure that we can continue to protect the NHS and its ability to cope. We must see a sustained fall in deaths. We must be sure that the infection rate is falling. We must overcome the operational and logistical challenges on testing and PPE.”

Here, Johnson focused more on how the community—‘we’–can fight the virus together. As described in the word cloud, Johnson often appealed to the people to take the measures seriously. Interestingly, he only seemed to include himself in this—as ‘we’–on specific occasions. Otherwise, he often spoke about the number of people who have fallen ill or died.

Taking a closer look at the transcripts, we find that Merkel’s ‘we’ often does not refer to the broad national community, as it does with Johnson, but rather to her and the representatives of the states and the European Union who had discussed certain matters. This reflects the couching of her executive decisions in communal processes involving the national federal system and the European Union:

“Ladies and gentlemen, over the past few days, we have been very intensively engaged in our meetings, and today we have discussed with each other via video conference where our country stands with regard to the epidemic emanating from the coronavirus. I would like to thank the states for pushing through the common line that we decided on, and that we agreed today to then also jointly shape the next phase. (April 15^th^ 2020)”

“A lot has happened in these months that we did not expect at our last European Council in February. One of them is that Italy has been affected in a very special way by the corona pandemic, very, very many people have lost their lives and Italy has made great sacrifices. We all agree that this pandemic has come upon us through no fault of anyone, and that is why it is now also our task to overcome the consequences of this pandemic. (July 13^th^ 2020)”

The above quote provides some insight into what this communal focus might mean to the public. The specific framing of “we” communicates how she was working together with other people—international collaborations, but also within Germany—and conveying the existence of a council of people advising her and helping her make decisions. Also noteworthy is the explicit reference that the pandemic is “no fault of anyone”.

The Swiss FC shows surprisingly low rates in we-talk, which is an unexpected result, as the council must appear as a collective—we expected them thus refer to themselves collectively. This result might be explained by a very frequent use of the word ‘federal council’ as we see in the word cloud. It appears instead that, rather than saying ‘we,’ the members generally referred to themselves as ‘the federal council’. This might indicate a psychological distancing from uncomfortable emotions. On March 20th 2020 the Swiss FC said:

“We must all now follow the measures. stay whenever possible at home. go out only in exceptional cases when you have to do urgent shopping when you have to go to the doctor pharmacy when you have to help other people or also for work when there is no home-office possible. this is really a very important moment that we are experiencing now. we really have to do a step together again and the federal council as I told or mentioned before wants whenever possible no curfew for Switzerland to our freedom belongs completely also the freedom of movement. but for that we need the whole population to join in now. to join in and stick to the recommendations. because ultimately it is not the exit barrier that protects us. you can also see that in other countries around us. what protects us is our behavior. that is our behavior. and we have to emphasize that once again. it is our behavior that decides whether the measures are successful and with that we can influence whether the intensive care units in the hospitals are overloaded or how many fall ill and die.”

We see here that ‘we’ references the general public and not themselves as the federal council specifically. Furthermore, individual responsibility as a commonsense imperative–see “generic you”—for communal protection is a clear focus. This is mirrored, somewhat in the word cloud, where we see ‘together’ as one of their most frequently used words.

### Emotional language

In a negative emotional tone, we see few noteworthy differences. Merkel showed the lowest negativity, while Johnson exhibited the greatest negative emotional content of all leaders—consistent with Johnson’s frequent focus on people falling ill or dying. Trump was significantly less negative than Johnson but showed no significant differences from the remaining political leaders. Again, Merkel and the Swiss FC spoke in a similar negative emotional tone, with no statistically significant differences.

Trump talked in the most positive emotional tone, while Johnson expressed the least positive emotional tone. Trump is significantly higher in his positive emotional tone than Johnson and the Swiss FC, but not Merkel. Johnson is significantly lower in positive emotional tone than Angela Merkel.

Trump's high positivity is consistent with the notion of populist leaders attempting to emotionally connect with people through an optimistic demeanor^22,30,42^. The nation itself was additional still in the midst of the ongoing Black Lives Matter movement^43^, with Trump being harshly criticized by many^44^. During such a time, the President may have opted to focus heavily on trying to maintain a positive impression of domestic situations. The following quote from Trump’s speech on June 5th 2020 paints a positive picture of national leadership reflected in the tone:

“I mean, we’ve had some great things. (Applause.) So — great. One of the many things we’ve done. But every person here today is playing a vital role in the greatest national industrial mobilization since World War Two. We’ve marshaled the full power of the U.S. government and U.S. industry to defeat the invisible enemy. And it is indeed an enemy. Should have been stopped in China. Came from China; should have been stopped in China. They didn’t do that.”

Many of Trump’s speeches are similar, wherein he discusses how well the United States was faring compared to other nations and their missteps or wrongdoings.

### Analytical thinking

In analytical speech, we see that Trump showed the lowest, and the Swiss FC the highest metrics. The Swiss FC's high scores in analytical speech further underline their distant tone in their speeches. Trump was significantly lower than all other political leaders. Boris Johnson and the Swiss FC were both significantly higher in analytical speech than Angela Merkel but showed no statistically significant difference from each other.

In their speech with the highest score—April 30th, 2020 of analytical thinking the Swiss FC spoke to the economic impact the pandemic had on the world and how they decided to invest money in supporting international efforts against the pandemic.

“The virus knows no borders and has consequences for the whole world. The crisis hits countries with low incomes, i.e. the poorest among us. Their situation will be even more precarious more precarious than it already is. The pandemic crisis is compounded by a severe economic crisis that threatens the livelihood of many people.”

On the other hand, on the 7th of March 2020, in the speech with Trump’s lowest value of analytical score, he discussed the high rates of virus testing as well as the efficacy of these measures:

“And the whole situation is — the testing has been amazing, actually. What they’ve been able to produce in such a short period of time. […] And they started working when they saw there was a problem in China. That was many weeks ago. So they saw there was something going on in China long before anybody even heard of it. That was actually before it was even in the print. They heard there was a problem in China. That’s when they started working on this, and that’s pretty incredible. That’s why we’re in good shape.”

### Authenticity

Trump's language exhibited the lowest degree of authenticity, while Angela Merkel’s language reflected relatively high levels of authenticity. The difference to the Swiss FC is not significant, though. In contrast, Trump’s language suggested significantly less authenticity than all other political leaders. Boris Johnson was significantly less authentic than Angela Merkel and the Swiss FC.

Merkel’s most authentic speech was on the 13th of July 2020. There, she said:

“Let me say this in the direction of the citizens of Germany: Germany, just like Italy and all the other member states of the European Union, has an interest in a functioning internal market. We have seen that when value chains are destroyed or no longer function, we are all equally affected. That is why we are now all equally responsible for finding a good way out of this situation for the European Union and for all member states.”

In his least authentic speech on August 5th 2020, Donald Trump was with the Governor of Arizona, again describing how well the pandemic has been handled by leadership:

“And he has done an incredible job on COVID, or COVID-19, or about 19 other names we can call it. It’s got probably more names than anything else you can think of. And he was hit very hard, and he’s — and he hit back even harder.”

## Discussion

This paper aimed to describe and compare the linguistic features of the political leaders of the USA, UK, Germany, and Switzerland during the beginning of the COVID-19 Pandemic. First, we looked at the most frequently used words by each highest executive power. We found many references to the people in the population in Trump’s, Johnson’s, and Merkel’s words, while Trump also showed words indicating his positivity, Johnson on his warnings against the virus, and Merkel’s collaborations with the European Union. The Swiss FC most often referenced themselves. When directly comparing the linguistic features of the political leaders, we found Trump to be the most positive, Johnson the most negative, Merkel to have the highest focus on community, and the Swiss FC to be the most analytic. We believe these differences mirror different attitudes, approaches, and strategies toward the pandemic. For example, our results show that Trump specifically promoted a positive view of the decisions made by political leadership, whereas Johnson adopted a focus on warning of the harmful and negative aspects of the virus itself . Merkel seems to have focused on conveying a sense of being ‘in this together’ and the Swiss FC kept a professional distance.

Our analyses did reveal, however, several unanticipated results. Contrary to our expectation was Trump’s low level of authenticity. In previous studies of Trump’s verbal behavior, he has been found to show high levels of authenticity, mirroring his very informal communication style^3,21,22^. One potential explanation would be that Trump’s rhetoric was perhaps not as fact-based as other leaders. While non-authentic language has been demonstrated to be related to deception^25^, we reiteratethat the authenticity metric calculated within our research is not necessarily a reflection of “honesty” or the truthfulness of the content of one’s speech. Furthermore, Trump’s previous, high-authenticity public speeches were not particularly fact-based either^45^. Alternatively, it could be argued that Trump may have been especially pressured to communicate content that was more carefully prepared and filtered than usual, reflecting a pressing need to convey carefully-crafted messaging to address the public’s pandemic-related needs. This is a speculation that calls for further research including e.g., a more detailed analysis of the political situation and information on the presidential communication requirements. His statements during the pandemic might have been adhering more to scripts than others in his career, due to more pressure to stick to the scripts than in other situations. While a politician's language commonly follows a script to some extent, it has been shown to reveal meaningful individual differences^4^; further research should investigate more closely possible differences between the more or less scripted language of political leaders, for example by comparing speeches with more spontaneous statements. On the other hand, the low levels of analytical language are typical for Trump and have been reported in previous studies^18,22^. Such lower levels in analytical speech might convey less perceived competence and cognitive ability^33^.

Following populist trends^46^, we expected Johnson to also express more emotions. In our analyses, Johnson showed relatively low levels of positive tone, but significantly higher levels of negative tone compared to the other leaders under study. Relative to Trump, then, we see that Johnson focused more acutely on negative emotions than positive ones. In line with discourse theory and other pandemic-related research, we suggest that the use of rhetoric to warn the population of the dangers of the pandemic and appeal to the public through negative emotions, such as fear, reflects a specific strategy to manage the public during the pandemic. Other recent studies have shown fear to be a key factor in adoption of COVID-19 safety measures^47^ and is also often employed by populist leaders^48^.

In Johnson’s word cloud and his specific use of ‘we-talk,’ his populist ideas are mirrored; Johnson uses ‘we’ to describe the people and himself. Contrastingly, Angela Merkel, who displayed the most use of ‘we-talk’, principally used it to describe a council of people and herself, mirroring Merkel's focus on and representation of the communal decisions of the federal system in Germany and the European Union. This is in strong contrast to what was seen in Trump’s rhetoric, which separates the USA from other nations in terms of the quality and efficacy of response measures. We suggest that the focus on community found in previous communications of Angela Merkel^19^ goes beyond the German people, but also represents her working in collaborations with other politicians and nations Although the communal orientation had a broader focus beyond the nation, Merkel’s popularity rose during the COVID-19 pandemic (https://www.politico.eu/europe-poll-of-polls/germany/).

The Swiss FC showed the highest mean levels of analytical language. Analytical language is described as coming across as more distant^22^. Considering that the Swiss FC must all stand in unison behind the decisions they make collectively—even if one specific member might have decided differently—it is understandable that these decisions were brought to the public with some distance. Such rationale stands in line with the Swiss FC referring to themselves in the third person and, further, not having either a strong positive or negative emotional tone in their speeches. Such a communication style can be understood to reflect one form of political culture within direct democratic system, where individual responsibility represents a high value while political personalities play a negligible role in the political system.

### Strengths and limitations

Our study has several strengths, including the direct comparison of text across multiple languages, cultures, and nations. There have been a large number of psychological and social studies regarding the COVID-19 pandemic, but few have directly compared different nations. The COVID-19 pandemic provided a unique opportunity to compare different political leaders, who are also in different political systems and thus hold different power, all while being in very similar situations.

Nevertheless, our study is not without limitations. First, we do not know the degree to which any given speech was planned, scripted, or rehearsed prior to delivery, nor the degree to which each member of any given political body shaped the specific wording or messaging ultimately delivered. This isa common limitation in studies on political leader language^4^. However, the language contained in political speeches can be thought of as not only a reflection of the psychology of individual leader themselves, but also as a snapshot of the political machinery that supports a leader’s administration, policies, motives, and goals^49^. Moreover, it is important to consider what word count-based indicators can provide and where there are limitations. Specific to our study, LIWC measures are not thought of as “content” indicators in a traditional sense, but rather as indicators of the linguistic style and underlying psychology of the speaker. Conceptually, the underlying assumption is that individuals’ linguistic style, beyond the content of what they are saying, serves as reflections of their psychological states and traits. This can be measured by the frequency of references to relevant linguistic concepts. Empirically, this assumption has been supported repeatedly^50^. Other linguistic features, representing more psychological dimensions and extracting relevant topics regarding the content of the speeches, would help to get more insight into messages of the leaders’ statements, their context, and the psychology behind them. We chose the scope of this study based on language dimensions that have been identified as meaningful but acknowledge that there are other dimensions of interest and different ways to measure them. Furthermore, our study focuses only on language markers not controlling for the role of personal and situational characteristics, such as ideological background, personality of the leaders, as well as case numbers or stringency of regulations in the corresponding nations. It is to assume that all this is reflected to a certain extent in language use, but a deeper understanding required further investigation. As an example, a recent study conducted with US state governors’ statements in early 2020 showed that over time, with higher COVID numbers the supposedly stricter regulation was reflected in more strict and negating language use^13^. This supports the notion that language actually reflects relevant aspects of the crisis as well as the leaders’ way to deal with it.

Lastly, we note that there were considerable differences in the number and length of speeches delivered by each leader during the investigated time period, with Trump holding many more public speeches relative to the others. The indicators used in this study are, however, representive of relatively stable stylistic components of language, capturing an aggregated and reliable facet of verbal behavior: language style^41^. In this regard, style-based indicators can be seen as reliable measures irrespective of speech length or frequency as investigated in this study, as each sample provided a sufficient size of language sample to provide reliable estimates^51^.

## Conclusion

Our study contributes to the field by closely describing and comparing different linguistic styles of political leaders during a time of collective crisis, opening the door for further studies which consider further contextual and individual factors that might influence a political leader’s language. In summary, our findings show that each leader approached this pandemic differently. Although similar measures were taken in each nation, leaders focused on different aspects in their public announcements. They also all exhibited different linguistic features and, moreover, looking at exemplary quotes, we see that the same linguistic features – such as the first personal plural pronoun ‘we’—can reflect different, equally important constructs and social formulations.

Almost by definition, the words that leaders use in response to a crisis reflects the crisis itself—not only the leaders’ values, personalities, and ideologies, but multiple contextual factors, including public need and expectations. Such factors are part of what makes language analysis so intriguing and promising for further research of communication during crisis. An analysis of speeches from the 4 leaders provides us insight into the leaders’ mental worlds and the context of their remarks. Their words provide insight into how Trump, Johnson, Merkel, and the Swiss FC understood and thought about the events of 2020. The strength of a text analytic approach as used in this study is that we are able to abstract language styles, summarizing the social psychological factors at play a clear, meaningful way, thereby contributing to a better understanding of the approaches each leader adopted in response to a truly global crisis.

## Data Availability

Data and R-codes are available on OSF, https://osf.io/zpwxh/?view_only=5fe19bc0d604470fbfe0de9d97c39ea2
